# Using Extreme Value Theory Approaches to Forecast the Probability of Outbreak of Highly Pathogenic Influenza in Zhejiang, China

**DOI:** 10.1371/journal.pone.0118521

**Published:** 2015-02-24

**Authors:** Jiangpeng Chen, Xun Lei, Li Zhang, Bin Peng

**Affiliations:** Department of Health Statistics and Information Management, School of Public Health and Management, Chongqing Medical University, Chongqing, China; University of Illinois at Chicago, UNITED STATES

## Abstract

**Background:**

Influenza is a contagious disease with high transmissibility to spread around the world with considerable morbidity and mortality and presents an enormous burden on worldwide public health. Few mathematical models can be used because influenza incidence data are generally not normally distributed. We developed a mathematical model using Extreme Value Theory (EVT) to forecast the probability of outbreak of highly pathogenic influenza.

**Methods:**

The incidence data of highly pathogenic influenza in Zhejiang province from April 2009 to November 2013 were retrieved from the website of Health and Family Planning Commission of Zhejiang Province. MATLAB “VIEM” toolbox was used to analyze data and modelling. In the present work, we used the Peak Over Threshold (POT) model, assuming the frequency as a Poisson process and the intensity to be Pareto distributed, to characterize the temporal variability of the long-term extreme incidence of highly pathogenic influenza in Zhejiang, China.

**Results:**

The skewness and kurtosis of the incidence of highly pathogenic influenza in Zhejiang between April 2009 and November 2013 were 4.49 and 21.12, which indicated a “fat tail” distribution. A QQ plot and a mean excess plot were used to further validate the features of the distribution. After determining the threshold, we modeled the extremes and estimated the shape parameter and scale parameter by the maximum likelihood method. The results showed that months in which the incidence of highly pathogenic influenza is about 4462/2286/1311/487 are predicted to occur once every five/three/two/one year, respectively.

**Conclusions:**

Despite the simplicity, the present study successfully offers the sound modeling strategy and a methodological avenue to implement forecasting of an epidemic in the midst of its course.

## Introduction

Influenza is a contagious disease with high transmissibility to spread around the world with considerable morbidity and mortality. Influenza occurs most frequently in late autumn, winter and early spring, reaching its peak prevalence mostly in winter. Seasonal influenza epidemics caused by influenza A and B viruses typically occur annually during winter in temperate regions and present an enormous burden on worldwide public health, resulting in around 3–5 million cases of severe illness and 250,000–500,000 deaths worldwide each year, according to the World Health Organization (WHO)[1].

In contrast to seasonal influenza, novel influenza A strains capable of sustained person-to-person transmission arise occasionally exacting an enormous toll on human health and economic well-being. Indeed, accurately forecasting the timing of influenza outbreaks, especially for highly pathogenic influenza, would provide greater lead time for preferential focus and response. Therefore, it is very important to have a good model describing influenza’s behavior and giving reliable predictions.

The prediction of influenza epidemics has long been the focus of attention in epidemiology and mathematical biology. Previous studies have used a variety of approaches to model influenza cases. Goldstein E *et al* [2] proposed a generalized linear model demonstrating how routine virologic and influenza-like illness (ILI) surveillance data can be used to quantify the dynamics of co-circulating influenza strains and generate short-term predictions of the relative epidemic sizes of each strain during the course of an influenza season in the United States. Soebiyanto RP *et al*. [3] employed an Autoregressive Integrated Moving Average (ARIMA) model along with climatic parameters to forecast the next influenza season. Mugglin AS *et al*. [4] used a Bayesian hierarchical approach for modelling of influenza epidemic dynamics in both time and space. Kim EK *et al*. [5] unexpectedly used Twitter, a free social networking service, to improve the accuracy of forecasting models by providing early warnings of influenza outbreak.

However, the application of Extreme Value Theory (EVT) remains relatively unexplored in the study of influenza forecasting. Other than our work, we have not acknowledged any EVT research conducted in the area of influenza forecasting to date. Only one study was related to the influenza, which applied the EVT to predict the distribution of extremes of mortality due to pneumonia and influenza for the >65 age group from the data of 1968–1998 in California and Texas [6].

The EVT is of interest in this study because of its potential to estimate the probability of extreme events from a relatively short period of observations. The history of EVT dates back to early work by Fisher and Tippett(1928) [7], Gnedenko(1943) [8], Gumbel(1958) [9] and Pickands(1975) [10], who lay the foundations of asymptotic theories and classic limit laws to describe the distributions of extremes.

The EVT has been proved to be a powerful tool to study extreme event distributions and widely used in many applications in multidisciplinary areas, such as value-at-risk estimation in finance [11]. EVT has been shown to be a very useful tool in estimating and predicting the extremal behavior of actuarial and financial products. However, the application of EVT in transportation engineering is relatively limited. One example by Lai Zheng [12] employs EVT to study the safety implications of lane change maneuvers in freeways. EVT satisfied the original intention of surrogate safety measures. Furthermore, EVT proposes a single dimension to measure the severity of surrogate events and to identify crashes, which fits within the classic safety hierarchy framework and abandons the assumption of fixed crash-to-surrogate ratio.

This study aims at extending the application of EVT to influenza prediction. In this paper, we use an EVT approach to model data on the monthly incidence of highly pathogenic influenza in Zhejiang province from April 2009 to November 2013. Eventually, several rules including different quantiles and the corresponding incidence of highly pathogenic influenza were established to predict the severity of the epidemic. Questions including when the outbreak will occur and what morbidity impact it will have in a certain place can thus be answered.

## Methods

### Data description

**Fig 1:**
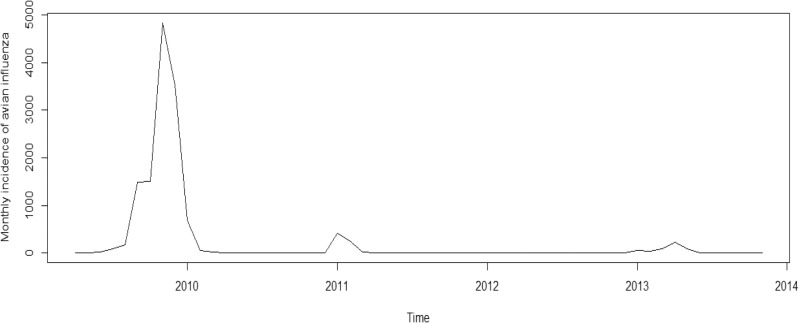
Monthly incidence of highly pathogenic influenza cases in Zhejiang province from April 2009 to November 2013. The vertical axis represents the monthly incidence of highly pathogenic influenza cases based on the provincial surveillance data. X-axis shows the corresponding time of each monthly surveillance data of highly pathogenic influenza.

To clearly explain the motivation in carrying out this study, the empirical data of highly pathogenic influenza incidence in Zhejiang province is presented. Fig. 1 shows the monthly reported numbers of influenza cases in Zhejiang province from April 2009 to November 2013. The data is publicly available on Zhejiang’s Health and Family Planning Commission website (http://www.zjwst.gov.cn) and typically released 1–2 week after the end of each month. During the period of interest, influenza A (H1N1) substantially dominated all other isolated influenza viruses. The data is solely laboratory confirmed influenza cases and does not include influenza-like-illness (ILI) cases. No ethics committee approval is required to obtain the data since it is publicly available. In addition, only count data is presented, no personal information is revealed, thereby maintaining confidentiality.

A detailed description of the incidence data is given as follows: Mean (244.09), Maximum (4834), Median (2.5), Minimum (0), Standard deviation (829.08), Skewness (4.49), Kurtosis (21.12).

### Estimation method (POT approach)

EVT is a powerful and yet fairly robust framework for studying the tail behavior of a distribution. There are two families of extreme value distributions corresponding to two approaches to sampling extreme events. The first is generalized extreme value (GEV) distribution which is used in the block maxima (BM) approach. The other is the generalized Pareto distribution (GPD) which is used in the peak over threshold (POT) approach. The BM approach assumes breaking up a sequence into blocks of size n (with n reasonably large), and extracting only the maximum observation *M*_i_(1,2,…,*n*) from each block regardless of whether the second largest event in a block exceeds the largest events of other blocks. Consequently, implementing the BM model requires a lot of data. In contrast to BM model, POT model uses a more natural way of determining whether an observation is extreme. All values greater than the given threshold are considered. Moreover, the largest incidence of influenza in a sporadic period can be so small that calling it an influenza outbreak can be misleading. The estimators from these extremes usually perform better than the BM approach for the same data set according to the previous studies [12–14]. Therefore, in this paper, we consider the POT approach to EVT.

An observation is treated as an extreme if an associated measurement exceeds a predetermined threshold. Suppose that χ_1_,χ_2_,…,χ_m_ is a sequence of independently and identically distributed random variables from an unknown distribution function *F*(χ) and m is the sample size. The distribution function of exceedances X over a threshold μ is defined by: $F_{\mu }\left(y\right)=\mathrm{Pr}\left\{Χ\text{-}\mu \leq y|Χ>\mu \right\}=\frac{F\left(y+\mu \right)\text{-}F\left(\mu \right)}{1-F\left(\mu \right)}$. With a high enough threshold μ, the conditional distribution *F*_μ_(*y*) can be approximated by a GPD. The cumulative distribution function of a GPD is given as follows:

$$G_{\xi ,\sigma }\left(\chi \right)=1-{\left(1+\frac{\xi \chi }{\sigma }\right)}^{-1/\xi }$$

Where σ > 0 is the scale parameter, and the - ∞ < ξ < +∞ is the shape parameter.

## Results

### Preliminary data analysis

**Fig 2:**
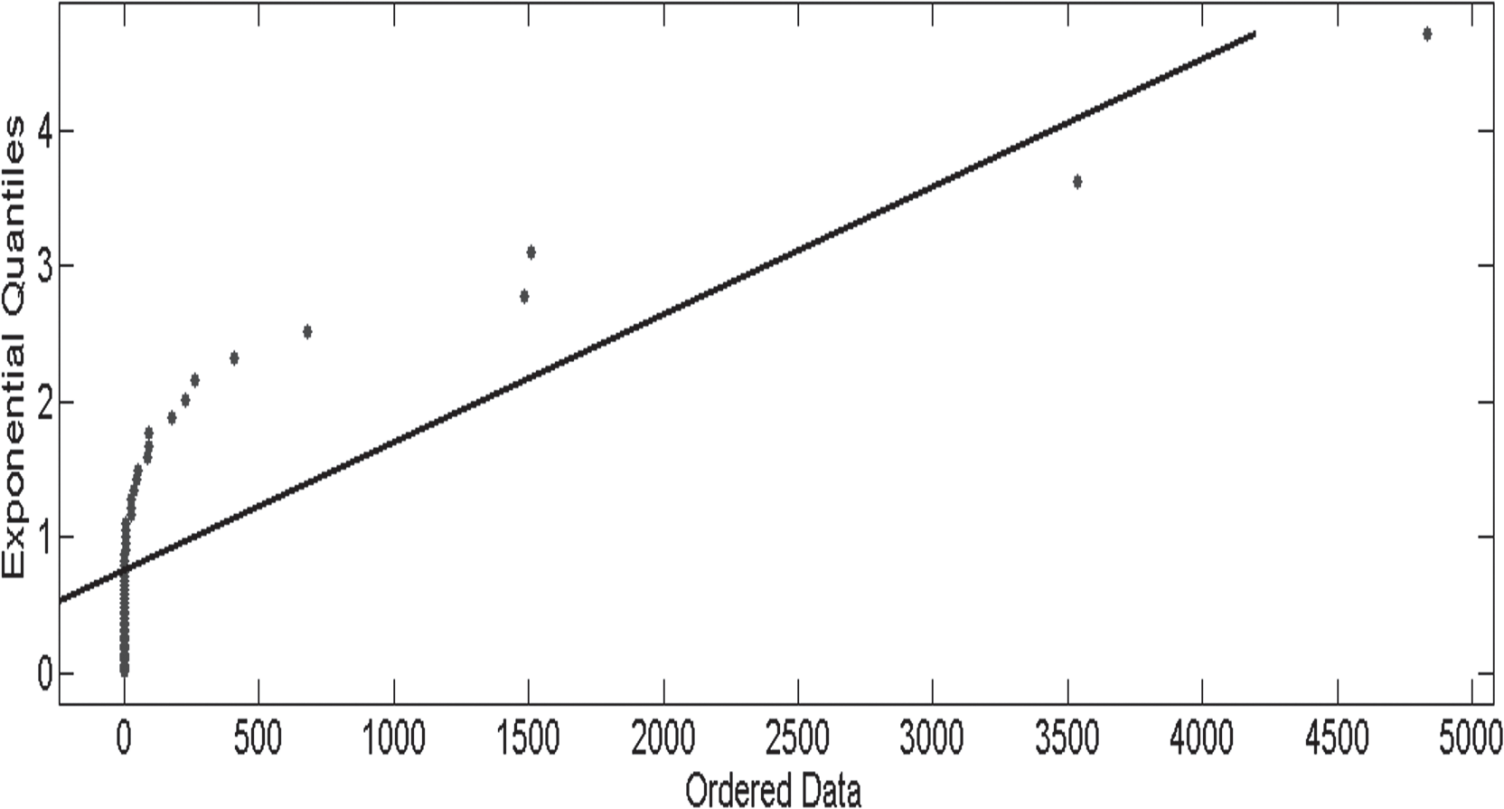
A QQ plot of the highly pathogenic influenza incidence data against standard exponential quantiles. Notice that a concave departure from the straight line in the QQ plot (as in this plot) is an indication of a heavy-tailed distributions.

In statistics, a quantile-quantile plot (QQ plot) is a convenient visual tool for examining whether a sample comes from a specific distribution. In the EVT and applications, the QQ plot is typically plotted against the exponential distribution to measure the fat-tailedness of a distribution. If the sample comes from the hypothesized distribution or a linear transformation of the hypothesized distribution, the QQ plot is linear. Notice that a concave departure from the straight line in the QQ plot (as in Fig. 2) is an indication of a heavy-tailed distributions, whereas a convex departure is an indication of a thin-tailed distributions.

**Fig 3:**
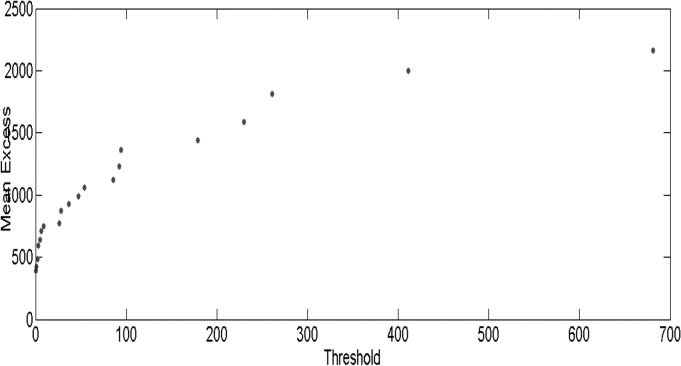
The sample mean excess of the highly pathogenic influenza incidence data against threshold values. The figure shows the empirical mean excess function has a positive slope above the threshold 9, which indicates the data follows the GPD with a positive shape parameter.

A second tool is the sample mean excess function (MEF), which is the sum of the excesses over the threshold μ divided by the number of data points that exceed the threshold μ. If the empirical MEF has a positive gradient above a certain threshold μ, it is an indication that the data follow the GPD with a positive shape parameter ξ (See Fig. 3).

### Choose the threshold

The sample size of the POT approach is determined by the selected threshold. Too high a threshold will likely lead to a large variance as there few observations over the threshold, while taking a lower threshold likely incorporates observations with ordinary values into extremes and then the asymptotic assumption becomes less valid.

The “Kurtosis method” (proposed by Pierre Patie) [15] was adopted to choose the threshold, which is relatively objective:

Step I: Calculate the sample Kurtosis *k_n_*, sample skewness μ*_n_* and sample variance $S_{n}^{2}$;
Step II: If *k_n_* ≥ 3, then remove the *X_i_* maximizing (*X_i_* - μ*_n_*)^2^ from the sample;
Step III: Repeat the first and second step until the Kurtosis less than 3;
Step IV: Choose the largest *X_i_* from the rest of the sample point as the threshold.

Thus, 9 is taken as the threshold of the highly pathogenic influenza incidence data and the number of exceedances is 18.

### Distribution and tail estimation

**Fig 4:**
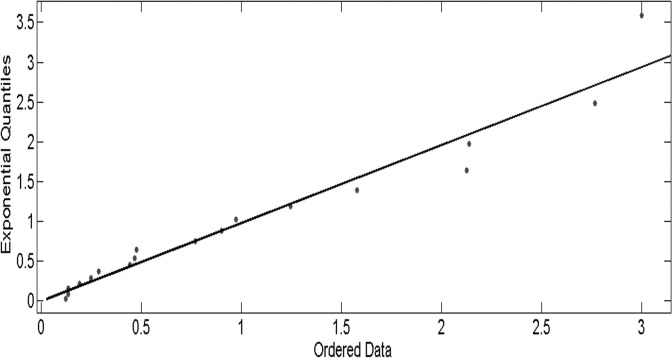
A QQ plot of residuals from GPD fit to the highly pathogenic influenza incidence data over threshold 9. The solid line corresponds to standard exponential quantiles. The approximate linear QQ plot indicates the residuals from GPD follow the standard exponential distribution.

The MATLAB toolbox “EVIM” provides a convenience for POT extreme analysis. We fit a Poisson point process to the highly pathogenic influenza incidence data with threshold parameter 9. Counts of all values over the threshold were described by the Poisson point process. The technique is also known as the POT method. Maximum likelihood estimation is one of the most popular techniques used to estimate the model parameters. Parameter estimates were obtained using the monthly incidence data. Maximum likelihood estimates of shape parameter and scale parameter were 1.3033 and 128.6390, respectively. Fig. 4 shows QQ plot of residuals from GPD fit to the highly pathogenic influenza incidence data over threshold 9, which indicates the data fits the distribution well.

### Predictions at the tail

We calculated quantile estimates and confidence intervals for high quantiles above the threshold in a GPD analysis. The confidence intervals are calculated from the quantiles of the empirical distributions. For example, the 95% confidence interval of estimated incidence of influenza is determined by taking the 2.5%-quantile as the lower bound and the 97.5%-quantile as the upper bound.

The estimated time interval between events of a similar size or intensity was calculated by the given quantile (generally expressed in percentages). For example, the return period of a flood might be 100 years; otherwise expressed as its probability of occurring being 1/100, or 1% in any one year. Take the 0.983 (quantile) for example, the probability of more than 4462 cases is 1.7%. Then we can calculate the return level (1/0.017) and transformed it into units of a year (the raw data's unit is months).

**Fig 5:**
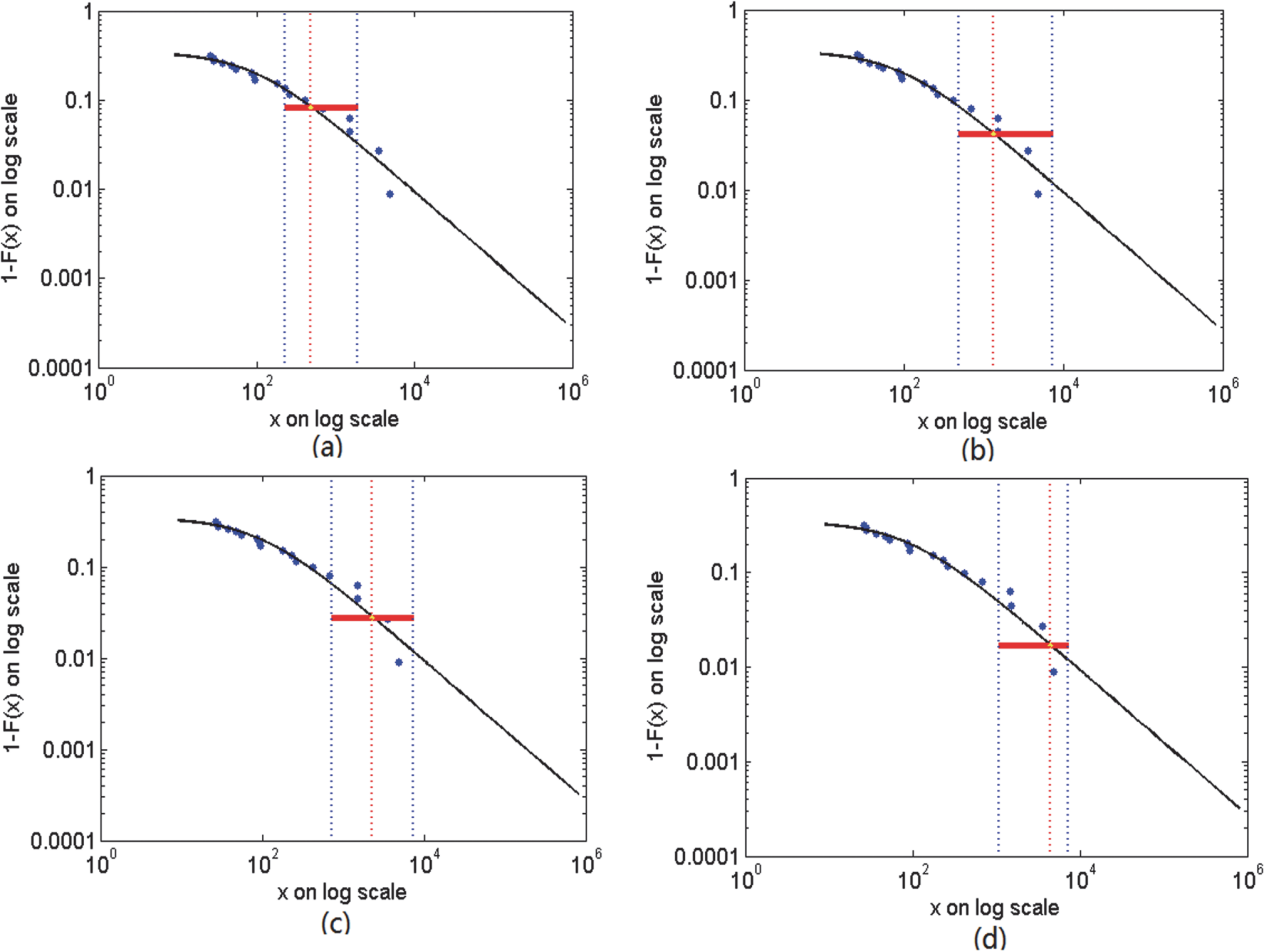
Point estimate at the tail. Panels a, b, c and d show the point estimation of monthly incidence of highly pathogenic influenza in Zhejiang province at different quantiles (0.917, 0.958, 0.972 and 0.983). Estimated tail is plotted as solid line, actual data in circles. Vertical dotted lines are estimated monthly incidence of highly pathogenic influenza in Zhejiang province at different quantiles (0.917, 0.958, 0.972 and 0.983) (middle dotted line), lower confidence level (left dotted line) and upper confidence level (right dotted line).

The predicted monthly incidence in Zhejiang province under different quantiles is displayed in Table 1. Fig. 5 shows the point estimate at the tail as well. Using the maximum likelihood estimates of parameters, the peak monthly incidence was predicted to range from 487 to 4462 as quantiles jumped to 0.983 from 0.917. According to the interpretation above, we could draw the conclusion that months in which the incidence of highly pathogenic influenza is about 4462/2286/1311/487 are predicted to occur once every five/three/two/one year, respectively. It is worth mentioning that the epidemic, the monthly incidence of highly pathogenic influenza is about 1311, would occur every two years, which is extremely useful for allocation of human resources for infectious diseases and prevention and treatment of highly pathogenic influenza.

**Table 1:** Estimation of the incidence of highly pathogenic influenza of Zhejiang province under diffident quantiles.

Quantile	Point estimation	95%CI	
		Lower bound	Upper bound
**0.983(Once in five years)**	4462	1087	7251
**0.972(Once in three years)**	2286	721	7251
**0.958(Once in two years)**	1311	483	7251
**0.917(Once in a year)**	487	228	1876

## Discussion

We used a mathematical method based on EVT for modelling and detecting extreme monthly incidence of highly pathogenic influenza in Zhejiang, China. The biggest advantage of the proposed model is its potential to describe and predict the extreme monthly incidence of highly pathogenic influenza with interpretable parameters under different quantiles. In addition, parameterization produces estimates that can be exploited to compute the extreme epidemic size. The attraction of the EVT based methods is that they can provide mathematically and statistically justifiable parametric models for the tail distribution, which can give reliable extrapolations beyond the range of the observed data [11]. To our knowledge, this is the first study utilizing EVT for estimating influenza activity in China or elsewhere in the world.

Note that the EVT basically captures the peaks of the influenza time series curve, thereby making it a good indicator for influenza activity in China. The influenza epidemic periodic mode of 2 years in the present study may be explained by the dynamics of pandemics (in 2009/2011 for H1N1 and 2013 for H7N9). The novel strains may evade existing antibody immunity and give rise to pandemic outbreaks. That is, after a major antigenic shift resulting in a pandemic, increasing numbers of members of a population come to possess the appropriate antibodies, and subsequent epidemics, decreasing in intensity and occurring at increasing intervals, are due to minor antigenic changes. Eventually another major antigenic shift occurs, and the cycle is repeated [16].

Despite the simplicity, the present study successfully offers the sound modeling strategy and a methodological avenue to implement forecasting of an epidemic in the midst of its course. It is anticipated that the present method based on EVT will contribute to further development in the field of prediction analysis of epidemics of influenza. The suitability of the EVT in the medical field was also demonstrated by Lee HC *et al* [6], though the BM model rather than the POT model used in our study was chosen in their research. Both of the researches applied the same theory to predict the distribution of extremes, and there are still some differences between the two studies. Our study was dedicated to forecasting the probability of outbreak of highly pathogenic influenza, while their study focused on modelling the mortality of the old people. Each study has its own strengths and limitations. Large amounts of mortality data allowed them to modelling based on the BM approach, and we further investigated the EVT and finally applied the POT model with the limited incidence data. In conclusion, the two studies proved it suitable to modelling in the medical field based on the EVT.

Nevertheless, this study has several limitations. Since only the incidence data of influenza in Zhejiang province was included in this study, the prediction outbreak probability can’t be applied to estimate the extreme value in other areas. In the extreme value modeling of natural phenomena such as sea level, wind speed, earthquake, as well as influenza pandemics, the validation task is virtually impossible because the prediction horizon is usually distant from the observed levels and the events of interest may have never existed in the past[17]. Thirdly, in order to have a relevant fit of the tails, we must consider a dataset which is large enough especially for the BM model. Although the POT model employed in this study is more suitable for small sample sizes than the BM model, more extensive surveillance of influenza is needed. Indeed, our dataset has several confounding factors, though all the surveillance data exist, such as are doctors more likely to order blood tests when presented with a patient with influenza like illness in November versus May.

As a first step applying EVT into influenza prediction analysis, the study shows that the application of EVT provides a promising approach for influenza prediction. Future work will explore more sophisticated measures to be applied to the EVT approach. In addition, determining a reasonable threshold is rather difficult in EVT. However, only the Kurtosis method is objective to select the threshold relative to other methods including Hill plot, Mean excess plot. Further work is still needed to account for the subjective issues in determining the threshold for the POT approach.

## Conclusions

This study used a mathematical model based on Extreme Value Theory to forecast the probability of outbreak of highly pathogenic influenza in Zhejiang, China. The influenza epidemic periodic mode of 2 years in the present study was basically consistent with the reality. Despite the simplicity, the present study successfully offers the sound modeling strategy and a methodological avenue to implement forecasting of an epidemic in the midst of its course. Nevertheless, several important topics need to be further investigated in the future work.
